# Complete genome sequence of *Rhodothermus marinus* type strain (R-10^T^)

**DOI:** 10.4056/sigs.46736

**Published:** 2009-12-29

**Authors:** Matt Nolan, Brian J. Tindall, Helga Pomrenke, Alla Lapidus, Alex Copeland, Tijana Glavina Del Rio, Susan Lucas, Feng Chen, Hope Tice, Jan-Fang Cheng, Elizabeth Saunders, Cliff Han, David Bruce, Lynne Goodwin, Patrick Chain, Sam Pitluck, Galina Ovchinikova, Amrita Pati, Natalia Ivanova, Konstantinos Mavromatis, Amy Chen, Krishna Palaniappan, Miriam Land, Loren Hauser, Yun-Juan Chang, Cynthia D. Jeffries, Thomas Brettin, Markus Göker, James Bristow, Jonathan A. Eisen, Victor Markowitz, Philip Hugenholtz, Nikos C. Kyrpides, Hans-Peter Klenk, John C. Detter

**Affiliations:** 1 DOE Joint Genome Institute, Walnut Creek, California, USA; 2 DSMZ - German Collection of Microorganisms and Cell Cultures GmbH, Braunschweig, Germany; 3 Los Alamos National Laboratory, Bioscience Division, Los Alamos, New Mexico, USA; 4 Biological Data Management and Technology Center, Lawrence Berkeley National Laboratory, Berkeley, California, USA; 5 Oak Ridge National Laboratory, Oak Ridge, Tennessee, USA; 6 University of California Davis Genome Center, Davis, California, USA

**Keywords:** thermophile, alkaliphile, nonmotile, non-sporulating, aerobic, heterotroph, *Sphingobacteriales*, *Rhodothermaceae*

## Abstract

*Rhodothermus marinus* Alfredsson *et al.* 1995 is the type species of the genus and is of phylogenetic interest because the *Rhodothermaceae* represent the deepest lineage in the phylum *Bacteroidetes. R. marinus* R-10^T^ is a Gram-negative, non-motile, non-spore-forming bacterium isolated from marine hot springs off the coast of Iceland. Strain R-10^T^ is strictly aerobic and requires slightly halophilic conditions for growth. Here we describe the features of this organism, together with the complete genome sequence, and annotation. This is the first complete genome sequence of the genus *Rhodothermus,* and only the second sequence from members of the family *Rhodothermaceae.* The 3,386,737 bp genome (including a 125 kb plasmid) with its 2914 protein-coding and 48 RNA genes is part of the *** G****enomic* *** E****ncyclopedia of* *** B****acteria and* *** A****rchaea * project.

## Introduction

Strain R-10^T^ (= DSM 4252 = ATCC 43812) is the type strain of *Rhodothermus marinus*, and the type species of the genus *Rhodothermus* [1], which would become the type genus of the not yet formally described family *‘Rhodothermaceae’*. *R. marinus* was described by Alfredsson *et al.* in 1988 as Gram-negative, non-motile and non-spore-forming rods [2]. The type strain, R-10^T^, was isolated from a submarine hot spring in Iceland [2]. The organism is of significant interest for its position in the tree of life within the small (two type strains) family ‘*Rhodothermaceae’* which has been placed in the phylum *Bacteroidetes* [3]. However, members of the genus *Rhodothermus* form a distinct evolutionary lineage together with members of the genus *Salinibacter* (one type strain). The lipid composition of members of these two genera are significantly different from other members of the phylum *Bacteroidetes* (see below) and further work is needed to decide whether these organisms should be retained in that phylum. Cell membranes are the most complex structures of the cell and differences in their polar lipid compositions have significance in the evolution and physiology of the cell. The genome sequence of *Salinibacter ruber* had been deciphered several years ago [4].

In addition to strain R-10^T^ there are several cultivated strains of the species known for with 16S rRNA sequences are publicly available, all of them isolated from diverse marine habitats: the Icelandic strains NR-29 (AF217498), R-18 [DSM 4253] (AF217495), NR-32 (AF217499), PRQ-34 (AF217496), PRQ-55 (AF217496) [2]; the Chinese isolates it-14 (EU214602), aa-1 (EU652039), YB16 (EU147499), D-3 (EF095715), WL (DQ812981), and YBD-3 (EU147498) from a survey of thermophilic bacteria and acidophiles from hot springs close to Xiamen Sea; the Japanese strains OKD7 (AF217493), which was initially described as the type strain of *R. obamensis*, but is now considered to be a member of the species *R. marinus* [1], was isolated from a shallow marine hydrothermal vent [2], and ‘*R. clarus*’ (AB252420), isolated from a terrestrial hot spring in Hyogo, Arima (unpublished); strains Ae70-SC-S (AB267450) from the Mariner Field in southern Lau Basin, and the unpublished strain PRI2902 ‘*R. profundus*’ (FJ624399) isolated from a deep sea hydrothermal vent at 2630 m depth on the East-Pacific Rise. Only three significantly similar sequences from uncultured phylotypes are known: clone Pol_B_97 (EF444679) from hot Greek spring waters, clone PmeaH2OA2 (EU249937) from seawater adjacent to a *Pacillopora meandrina* coral colony at Palmyra Atoll, and clone SSE_L4_E01(EU635901) from 77°C warm sediments of hot springs in Nevada. No closely related sequences (over 86% sequence similarity) that could be directly linked to the species *R. marinus* were detected from environmental samples or genomic surveys (June 2009).

Here we present a summary classification and a set of features for *R. marinus* strain R-10^T^ together with the description of the complete genome sequencing and annotation.

### Classification and features

**Figure 1:**
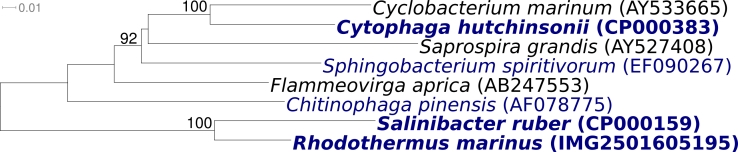
Phylogenetic tree highlighting the position of *R. marinus* strain R-10^T^ relative to *S. ruber*, the second species within the ‘*Rhodothermaceaea*’. Rooting was done with the other type strains of the order ‘*Sphingobacteriales’* (which, in our opinion, is not adequately defined; see the discussion above). The tree was inferred from 1,396 aligned characters [5,6] of the 16S rRNA sequence under the maximum likelihood criterion [7]. The branches are scaled in terms of the expected number of substitutions per site. Numbers above branches are support values from 1,000 bootstrap replicates if larger than 60%. Strains with a genome sequencing project registered in GOLD [8] are printed in blue; published genomes in bold.

Figure 1 shows the phylogenetic neighborhood of *R. marinus* strain R-10^T^ in a 16S rRNA based tree. The sole 16S rRNA gene sequence in the genome of strain R-10^T^ is identical with the recently published 16S rRNA gene sequence generated from DSM 4252 (AF217494) [1]. This is not a trivial statement, given the frequent and often significant differences detected when comparing sequences derived from GEBA genomes with ‘ancient’ 16S rRNA sequences deposited in the INSDC public repositories and listed in the Taxonomic Outline of *Bacteria* and *Archaea* [3]. For example, the sequence presented here differs by one nucleotide from the originally deposited sequence for strain R-10^T^ (X80994) [1] generated more than a decade ago, which also contains three ambiguous base calls. The difference between the genome data and the previously reported 16S rRNA gene sequences is most likely due to sequencing errors in the previously reported sequence data.

**Figure 2:**
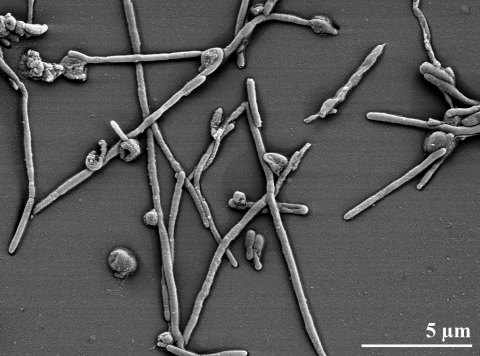
Scanning electron micrograph of *R. marinus* strain R-10^T^ (Manfred Rohde, Helmholtz Centre for Infection Research (HZI), Braunschweig)

*R. marinus* cells are rod-shaped, about 0.5 µm in diameter and 2-2.5 µm long [2] (Table 1, and Figure 2). Spores, flagella, and lipid granules were not observed [2], but a slime capsule is formed when grown in carbohydrate-rich media [2]. Colonies of strain R-10^T^ are convex and reddish-colored, containing a carotinoid pigment [2]. The strain is obligately aerobic and moderately halophilic [2]. Optimal growth is at 65°C (T_max_ 77°C), pH 7, with about 2% (w/v) NaCl. *Rhodothermus* strains can be distinguished from members of the genus *Thermus* by their requirement for higher concentration of NaCl in the growth medium, which is not tolerated by *Thermus* strains [2]. Cells are oxidase negative, but catalase positive. Nitrate reduction was not detected, and sugars are not fermented anaerobically [2].

**Table 1:** Classification and general features of *R. marinus* R-10^T^ in accordance with the MIGS recommendations [9]

**MIGS ID**	**Property**	**Term**	**Evidence code**
	Classification	Domain *Bacteria*	TAS [10]
		Phylum *Bacteroidetes*	TAS [3]
		Class not adequately defined	NAS
		Order not adequately defined	NAS
		Family ‘*Rhodothermaceae*’	NAS
		Genus *Rhodothermus*	TAS [1]
		Species *Rhodothermus marinus*	TAS [1,2]
		Type strain R-10	
	Gram stain	negative	TAS [2]
	Cell shape	rods	TAS [2]
	Motility	nonmotile	TAS [2]
	Sporulation	non-sporulating	TAS [2]
	Temperature range	thermophile, 65-80°C	TAS [2]
	Optimum temperature	65°C	TAS [2]
	Salinity	halophile, requires 0.5-2% (w/v) NaCl	TAS [2]
MIGS-22	Oxygen requirement	aerobic	TAS [2]
	Carbon source	glucose, maltose, galactose, lactose, raffinose, maltose, pyruvate, acetate, gelatin	TAS [1]
	Energy source	heterotroph	TAS [2]
MIGS-6	Habitat	marine hot spring	TAS [2]
MIGS-15	Biotic relationship	Free living	
MIGS-14	Pathogenicity	none	NAS
	Biosafety level	1	TAS [11]
	Isolation	submarine alkaline hot spring	TAS [2]
MIGS-4	Geographic location	Reykajanes, Isafjardardjup Bay, off cost of Iceland	TAS [2]
MIGS-5	Sample collection time	about 1988	TAS [2]
MIGS-4.1 MIGS-4.2	Latitude – Longitude	+63.88, -22.5	NAS
MIGS-4.3	Depth	2-3 m below sea level	TAS [2]
MIGS-4.4	Altitude	sea level	TAS [2]

Evidence codes - IDA: Inferred from Direct Assay (first time in publication); TAS: Traceable Author Statement (i.e., a direct report exists in the literature); NAS: Non-traceable Author Statement (i.e., not directly observed for the living, isolated sample, but based on a generally accepted property for the species, or anecdotal evidence). These evidence codes are from the Gene Ontology project [12]. If the evidence code is IDA, then the property was observed for a living isolate by one of the authors, or an expert mentioned in the acknowledgements.

*R. marinus* is able to utilize several common sugars (glucose, galactose, sucrose, lactose, raffinose, maltose), but glutamate and aspartate were the only amino acids used by most strains belonging to the species [2]. Genes for many biotechnologically interesting, predominantly carbohydrate metabolism-related enzymes have been described for strains belonging to the species [13-18]. A gene transfer system for *R. marinus* has been established [19], and a proprietary-hold genome sequence of a non-disclosed *R. marinus* strain has been generated at Prokaria, Iceland [8].

The composition of the peptidoglycan of *R. marinus* R-10^T^ is unknown. The sole respiratory lipoquinones are menaquinones, with MK-7 predominating. Polar lipids are largely phospholipids, a feature significantly different from aerobic members of the phylum *Bacteroidetes*. The major polar lipids are phosphatidylglycerol, phosphatidylethanolamine, two unidentified phospholipids and two unidentified lipids [20,21]. Glycolipids are minor components [20]. The cellular fatty acid profile of strain *R. marinus* R-10^T^ is dominated by branched-chain acids with mostly odd-chain lengths: iso-C_17_ (25.3%), anteiso-C_15_ (20.0%), anteiso-C_17_ (19.9%), and iso-C_15_ (8.1%), and some even-chain acids: iso-C_16_ (11.4%), iso-C_18_ (5.1%). Differences in the fatty acid composition reported by Tindall [20] and Nunes *et al.* [21] can be traced to differences in the growth conditions [22]. Straight chain acids constitute only a minority within the fatty acids spectrum: C_16_ (6.1%) [1].

## Genome sequencing and annotation information

### Genome project history

This organism was selected for sequencing on the basis of its phylogenetic position, and is part of the *** G****enomic* *** E****ncyclopedia of* *** B****acteria and* *** A****rchaea * project. The genome project is deposited in the Genomes OnLine Database [8] and the complete genome sequence in GenBank. Sequencing, finishing and annotation were performed by the DOE Joint Genome Institute (JGI). A summary of the project information is shown in Table 2.

**Table 2:** Genome sequencing project information

**MIGS ID**	**Property**	**Term**
MIGS-31	Finishing quality	Finished
MIGS-28	Libraries used	Three genomic libraries: two Sanger libraries - 8 kb pMCL200 and fosmid pcc1Fos – and one 454 pyrosequence standard library
MIGS-29	Sequencing platforms	ABI3730, 454 GS FLX
MIGS-31.2	Sequencing coverage	8.8x Sanger; 23.8 × pyrosequence
MIGS-30	Assemblers	Newbler version 1.1.02.15, phrap
MIGS-32	Gene calling method	Prodigal 1.4, GenePRIMP
	INSDC ID	CP001807 (chromosome) CP001808 (plasmid)
	Genbank Date of Release	2009/11/16
	GOLD ID	Gc01147
	NCBI project ID	29281
	Database: IMG-GEBA	2501533216
MIGS-13	Source material identifier	DSM 4252
	Project relevance	Tree of Life, GEBA

### Growth conditions and DNA isolation

*R. marinus* R-10^T^, DSM 4252, was grown in DSMZ medium 630 (modified *Thermus* 162 medium) plus 1% NaCl [23] at 65°C. DNA was isolated from 1-1.5 g of cell paste using Qiagen Genomic 500 DNA Kit (Qiagen, Hilden, Germany) with a modification of the standard protocol, LALMP, according to Wu *et al*. [24].

## Genome sequencing and assembly

The genome was sequenced using a combination of Sanger and 454 sequencing platforms. All general aspects of library construction and sequencing performed at the JGI can be found at http://www.jgi.doe.gov/. 454 pyrosequencing reads were assembled using the Newbler assembler version 1.1.02.15 (Roche). Large Newbler contigs were broken into 3,764 overlapping fragments of 1,000 bp and entered into assembly as pseudo-reads. The sequences were assigned quality scores based on Newbler consensus q-scores with modifications to account for overlap redundancy and to adjust inflated q-scores. A hybrid 454/Sanger assembly was made using the parallel phrap assembler (High Performance Software, LLC). Possible mis-assemblies were corrected with Dupfinisher or transposon bombing of bridging clones [25]. Gaps between contigs were closed by editing in Consed, custom primer walk or PCR amplification.  A total of 144 Sanger finishing reads were produced to close gaps, to resolve repetitive regions, and to raise the quality of the finished sequence. The error rate of the completed genome sequence is less than 1 in 100,000. The final assembly consists of 27,590 Sanger and 432,032 pyrosequence reads. Together all sequence types provided 32.6 x coverage of the genome.

### Genome annotation

Genes were identified using Prodigal [26] as part of the Oak Ridge National Laboratory genome annotation pipeline, followed by a round of manual curation using the JGI GenePRIMP pipeline [27]. The predicted CDSs were translated and used to search the National Center for Biotechnology Information (NCBI) nonredundant database, UniProt, TIGRFam, Pfam, PRIAM, KEGG, COG, and InterPro databases. Additional gene prediction analysis and manual functional annotation was performed within the Integrated Microbial Genomes Expert Review (IMG-ER) platform [28].

### Genome properties

**Figure 3:**
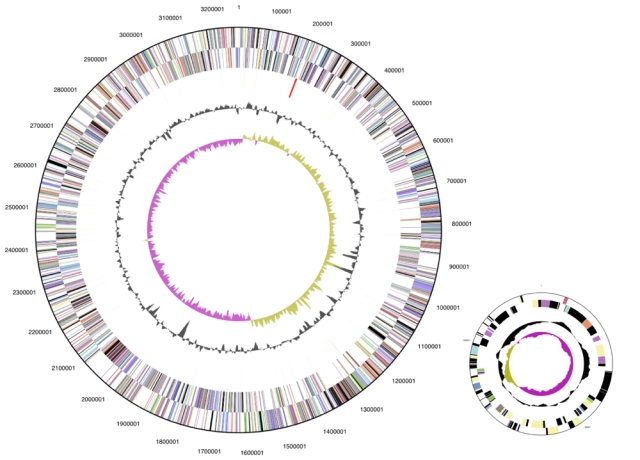
Graphical circular map of the genome (lower right: 125 kbp plasmid, not drawn to scale). From outside to the center: Genes on forward strand (color by COG categories), Genes on reverse strand (color by COG categories), RNA genes (tRNAs green, rRNAs red, other RNAs black), GC content, GC skew.

The genome is 3,386,737 bp long and comprises one main circular chromosome and one circular plasmid (125 kbp) with a 64.3% GC content. (Table 3 and Figure 3). Of the 2,962 genes predicted, 2,914 were protein coding genes, and 48 RNAs. In addition, 51 pseudogenes were also identified. The majority of the genes (71.6%) were assigned with a putative function while the remaining ones are annotated as hypothetical proteins. The distribution of genes into COGs functional categories is presented in Table 4.

**Table 3:** Genome Statistics

**Attribute**	**Value**	**% of Total**
Genome size (bp)	3,386,737	100.00%
DNA Coding region (bp)	3,133,821	92.53%
DNA G+C content (bp)	2,177,804	64.30%
Number of replicons	2	
Extrachromosomal elements	1	
Total genes	2,962	100.00%
RNA genes	48	1.62%
rRNA operons	1	
Protein-coding genes	2,914	98.38%
Pseudo genes	51	1.72%
Genes with function prediction	2,122	71.64%
Genes in paralog clusters	291	9.82%
Genes assigned to COGs	2,127	71.81%
Genes assigned Pfam domains	2,173	73.36%
Genes with signal peptides	710	23.97%
Genes with transmembrane helices	647	21.84%
CRISPR repeats	10	

**Table 4:** Number of genes associated with the general COG functional categories

**Code**	**value**	**% age**	**Description**
J	138	4.7	Translation, ribosomal structure and biogenesis
A	1	0.0	RNA processing and modification
K	132	4.5	Transcription
L	120	4.1	Replication, recombination and repair
B	2	0.1	Chromatin structure and dynamics
D	27	0.9	Cell cycle control, mitosis and meiosis
Y	0	0.0	Nuclear structure
V	43	1.5	Defense mechanisms
T	119	4.1	Signal transduction mechanisms
M	152	5.2	Cell wall/membrane biogenesis
N	51	1.8	Cell motility
Z	0	0.0	Cytoskeleton
W	0	0.0	Extracellular structures
U	61	1.1	Intracellular trafficking and secretion
O	95	3.3	Posttranslational modification, protein turnover, chaperones
C	133	4.6	Energy production and conversion
G	155	5.3	Carbohydrate transport and metabolism
E	188	6.5	Amino acid transport and metabolism
F	67	2.3	Nucleotide transport and metabolism
H	117	4.0	Coenzyme transport and metabolism
I	80	2.8	Lipid transport and metabolism
P	126	4.3	Inorganic ion transport and metabolism
Q	60	2.1	Secondary metabolites biosynthesis, transport and catabolism
R	288	9.9	General function prediction only
S	176	6.0	Function unknown
-	787	26.1	Not in COGs
